# Full genome characterization of a Kenyan G8P[14] rotavirus strain suggests artiodactyl-to-human zoonotic transmission

**DOI:** 10.1186/s41182-025-00759-9

**Published:** 2025-06-16

**Authors:** Ernest Apondi Wandera, Yuki Akari, Carlene Sang, Pamela Njugu, Samoel Ashimosi Khamadi, Sebastian Musundi, Maurine Mumo Mutua, Saori Fukuda, Takayuki Murata, Shingo Inoue, Satoshi Kaneko, James Nyangao, Satoshi Komoto

**Affiliations:** 1 Centre for Virus Research, Kenya Medical Research Institute (KEMRI), P.O. Box 54840-00200, Nairobi, Kenya; 2 Innovation and Technology Transfer Division, KEMRI, P.O. Box 54840-00200, Nairobi, Kenya; 3 Kenya Research Station, Nagasaki University Institute of Tropical Medicine (NUITM)-KEMRI, P.O. Box 19993-00202, Nairobi, Kenya; 4 Division of One Health, Research Center for GLOBAL and LOCAL Infectious Diseases (RCGLID), Oita University, Yufu, Oita 879-5593 Japan; 5 KEMRI Wellcome Trust Research Programme, KEMRI, P.O. Box 230, Nairobi, Kenya; 6 Department of Virology, Fujita Health University School of Medicine, Toyoake, Aichi 470-1192 Japan; 7 Center for Infectious Disease Research, Research Promotion Headquarters, Fujita Health University, Toyoake, Aichi 470-1192 Japan

**Keywords:** Group A rotavirus, Full genome-based analysis, G8P[14] strains, Artiodactyls, Interspecies transmission, Kenya, Africa

## Abstract

**Background:**

Rotavirus infections are a major cause of severe gastroenteritis in children. Human rotavirus strains with the unconventional G8P[14] genotype have sporadically been detected in diarrheic patients in different parts of the world. However, full genomes of only two human G8P[14] strains from Africa (North Africa) have been sequenced, and the origin and evolutionary patterns of African G8P[14] strains remain to be elucidated.

**Methods:**

In this study, we sequenced the full genome of an African G8P[14] strain (RVA/Human-wt/KEN/A75/2000/G8P[14]) identified in archival stool samples from a diarrheic child in Kenya.

**Results:**

Full genome-based analysis of strain A75 revealed a unique genogroup constellation, G8-P[14]-I2-R2-C2-M2-A11-N2-T6-E2-H3, with the I2-R2-C2-M2-A11-N2-T6-E2-H3 part being common among rotavirus strains from artiodactyls such as cattle. Phylogenetic analysis showed that all the 11 genomic segments of strain A75 are closely related to segments found in artiodactyl rotavirus strains, and likely strain A75 derived from spillover transmission of an artiodactyl rotavirus strain to humans.

**Conclusion:**

This is the first report on a full genome-based characterization of a human G8P[14] strain from East Africa. This study demonstrates the diversity of human G8P[14] strains in Africa and contributes to the elucidation of their spreading and evolution, which includes zoonotic transmission from artiodactyls.

**Supplementary Information:**

The online version contains supplementary material available at 10.1186/s41182-025-00759-9.

## Background

Group A rotavirus (RVA), belonging to the *Sedoreoviridae* family, is one of the major causes of severe gastroenteritis in children and young animals worldwide. In humans, RVA disease is associated with high morbidity and mortality, accounting for an estimated annual 128,500–215,000 deaths in children < 5 years of age, over half of which occur in African countries [1, 2]. Despite this high burden of RVA disease in Africa, data on the genotype diversity of its circulating RVA strains are limited, in particular for sub-Saharan African countries [3–5].

The mature RVA particle is a non-enveloped icosahedron with three layers, encapsulating an 11-segment double-stranded (ds)RNA genome [6]. RVA strains generally only infect a narrow range of host species, but occasional wider interspecies transmissions—sometimes involving segment reassortment—have been reported, contributing to the high genetic diversity of this medical and veterinary important virus [7–13]. RVA strains are classified based on the outer capsid proteins VP7 and VP4, which each induce neutralizing antibodies. VP7 is a glycoprotein, determined by the G genotype, and VP4 is a protease-sensitive protein, determined by the P genotype, and, to date, 42 G and 58 P genotypes have been recognized (https://rega.kuleuven.be/cev/viralmetagenomics/virus-classification/rcwg). Within individual host species, specific G and P genotypes are dominant, and in human RVAs (HuRVAs) these globally are G1-G4, G9, and G12 along with P[4], P[6], and P[8]. In Africa, G8 and P[6] genotypes, in various genomic constellations, are common among HuRVA genotypes [14–17].

G8 strains, representing one of the most common G genotypes detected in artiodactyls (even-toed ungulates) such as cattle [11, 18, 19], were first identified in cattle in 1965 [20]. G8 strains have been detected in humans in combination with either a P[1], P[4], P[6], P[8], P[10], or P[14] genotype [21–26]. P[14] strains, representing the P genotype commonly found in rabbits and artiodactyls, have also been detected in diarrheic children mainly in combination with the G6 genotype and, to a lesser extent, with the G1, G3, G8, or G10 genotype [27, 28]. The first G8P[14] strain, HAL1166, was detected in a diarrheic child in Finland in 1986 [29, 30], and subsequently in Australia [31, 32], Denmark [33], Egypt [34, 35], Greece [36], Guatemala [37], Hungary [38–40], Italy [41, 42], Japan [43, 44], Kenya [45, 46], Morocco [47], Slovenia [48], Taiwan [49], and the United States [50]. In host species other than humans, G8P[14] strains have been detected predominantly in artiodactyls: alpaca in Peru [51, 52], cattle in India [53] and Japan [54], guanaco in Argentina [55], roe deer in Slovenia [56], sheep in Spain [57], and vicuña in Argentina [58]. From Kenya, the detection of only one G8P[14] strain from a diarrheic child was reported [45, 46], without full-length sequence data of any genomic segments were obtained in the study. During a retrospective analysis of stool samples collected from Kenyan children < 5 years old with diarrhea in 2000–2002, and previously failed to identify G and/or P genotype(s), we detected another human G8P[14] strain, A75, from a diarrheic child in a total of 285 RVA-positive stool specimens. Among the 285 RVA-positive specimens, a total of 108 samples were randomly selected and subjected to G and P genotyping using the semi-nested RT-PCR method [59] with G-specific (G1–G4, G8, and G9) [15, 59, 60] and P-specific (P[4], P[6], and P[8]–P[10]) primers [61, 62]. In brief, of the strains that could be genotyped, G1P[8] strains were predominant (*n* = 20; 18.5%), followed by G2P[4] strains (*n* = 7; 6.5%). In addition, G8 (G8P[4] (*n* = 4), G8P[6] (*n* = 5), and G8PNT (PNT, P non-typeable) (*n* = 1)) (*n* = 10; 9.3%) and G9 (G9P[4] (*n* = 1), G9P[6] (*n* = 2), and G9P[8] (*n* = 5)) (*n* = 8; 7.4%) strains were detected in similar proportions.

Full genome-based analysis is important for understanding the origin of an RVA strain, and for tracing its path of evolution [9, 57]. To date, full genomes of several G8P[14] strains from humans and animals across different parts of the world have been sequenced and analyzed, revealing a conserved unique genotype constellation of the non-G/P segments commonly shared with RVA strains from artiodactyls: I2-R2-C2-M2-A3/A11-N2-T6-E2/E12-H3 [26–28, 39, 40, 63, 64]. The human P[14] strains are speculated to have originated from interspecies transmission from artiodactyls [27]. From Africa, full genome-based analysis of only one human G8P[14] strain, Moroccan ma31, has been reported in article format to date, providing evidence of its unique artiodactyl genomic backbone [47]. In addition, the full genome sequence of an Egyptian human G8P[14] strain, AS970, has been directly submitted to the DDBJ and EMBL/GenBank data libraries. However, a broader analysis of African G8P[14] strains remains necessary to understand their spread and history on the continent. Because strains ma31 and AS970 were isolated from North Africa, in the present study we sequenced and analyzed the full genome of Kenyan G8P[14] strain A75, which we had previously identified as G8PNT via PCR in an analysis of a stool sample collected from a Kenyan children < 5 years old with diarrhea [65]. Our detailed sequence analysis revealed a close relationship between A75 and artiodactyl RVA strains.

## Methods

### Case report

Strain A75 was identified in a stool specimen obtained from an 8-month-old female child admitted to the Aga Khan University Hospital, Nairobi in the year 2000 during the hospital-based HuRVA strain surveillance program in Nairobi and Kisumu counties in 2000–2002, which involved collecting a total of 285 RVA-positive stool specimens [65]. The child was presenting with severe acute gastroenteritis, having experienced diarrhea characterized by more than three episodes of looser than normal or watery stool in a 24-h period prior to admission, coupled with episodes of vomiting, fever, and severe dehydration. Prior to the sampling, the diarrheic child had never received HuRVA vaccinations. The child had come from a socio-economically affluent family who lived in Kiambu County, Central Kenya on a farm that practiced crop cultivation and animal rearing. Kiambu County borders Nairobi and Kajiado Counties to the South, Machakos County to the East, Murang’a County to the North and North East, Nyandarua County to the North West, and Nakuru County to the West. 

### Virus strain

Fecal specimen was obtained from the study child following an informed consent from the parent and shipped to the Centre for Virus Research laboratories at the KEMRI for processing. The sample was screened for RVAs using enzyme-linked immunosorbent assay and genotyped using multiplex semi-nested RT-PCR [65]. The stool sample was kept at − 80 °C until full genome sequencing that was conducted later.

### Viral genomic dsRNA extraction, cDNA library construction, and next-generation MiSeq sequencing

RVA genomic dsRNA of strain A75 was extracted from 10% stool suspensions using a QIAamp Viral RNA Mini Kit (Qiagen), and the dsRNA was subjected to direct Illumina MiSeq next-generation sequencing as described previously [22, 66]. In brief, a 200 bp fragment library ligated with bar-coded adapters was generated using an NEBNext Ultra II RNA Library Prep Kit for Illumina (New England Biolabs) according to the manufacturer’s instructions. The cDNA library was purified using NEBNext Sample Purification Beads (New England Biolabs). After evaluating the quality and quantity of the purified cDNA library, 151-cycle paired-end nucleotide sequencing was performed on a MiSeq sequencer (Illumina) using a MiSeq Reagent Kit v2 (Illumina). MiSeq sequence data were analyzed using CLC Genomics Workbench v8.0.1 (CLC Bio). Contigs were assembled from the yielded sequence data, after trimming, by de novo assembly. Using the assembled contigs as query sequences, the Basic Local Alignment Search Tool (BLAST) (https://blast.ncbi.nlm.nih.gov/Blast.cgi?PROGRAM=blastn&PAGE_TYPE=BlastSearch&LINK_LOC=blasthome) function on the National Center for Biotechnology Information (NCBI) was used to screen the GenBank non-redundant nucleotide database for assessing which contigs included full-length sequences of individual genomic segments of strain A75. To further polish the contigs, sequence reads of individual genes were mapped back to the assembled contigs. The RVA nucleotide sequences were translated into amino acid sequences using GENETYX v11 (GENETYX).

### HuRVA genotyping and phylogenetic analyses

The genotype of each of the 11 genomic segments of strain A75 was determined using the Rotavirus A Genotyping Tool v0.1 on Rijksinstituut voor Volksgezondheid en Milieu (RIVM) (https://www.rivm.nl/mpf/typingtool/rotavirusa/) and Nucleotide BLAST. The percentage nucleotide sequence identities of strain A75 to the reference RVA strains were calculated using the FASTA program [67] with GENETYX v11.

Multiple alignment of each genomic segment was conducted with MUSCLE [68], which is included in the MEGA software package, version 7.0.26 [69]. Maximum-likelihood phylogenetic trees were constructed for all 11 genomic segments. The best substitution models for the 11 genomic segments were selected based on the corrected Akaike information criterion (AIC_C_) value, as implemented in MEGA7.0.26. The AIC_C_ approach offers important advantages over the hierarchical likelihood ratio tests, namely, it can simultaneously compare multiple nested or non-nested models, accounts for model selection uncertainly, and allows for model-averaged inference [70, 71]. The models used in this study were Tamura 3-parameter (T92) + gamma distributed (G) + invariable sites (I) (VP7, VP6, NSP4, and NSP5), General Time Reversible (GTR) + G + I (VP4, VP2, VP3, and NSP1), Tamura-Nei (TN93) + G + I (VP1), T92 + G (NSP2), and TN93 + G (NSP3). The reliability of the branching orders was estimated using 1,000 bootstrap replicates. The mVISTA online platform was used to visualize the sequence similarities of the concatenated whole genome of strain A75 with those of artiodactyl and artiodactyl-like human strains as references [72]. Amino acids alignments on the 11 RVA proteins (VP7, VP4, VP6, VP1-VP3, and NSP-NSP5) were performed with CLUSTAL W [73], which is included in the GENETYX v11.

### Nucleotide sequence accession numbers

The nucleotide sequence data obtained in this study have been deposited in the DDBJ and EMBL/GenBank data libraries. The accession numbers for the nucleotide sequences of the VP1–VP4, VP6, VP7, and NSP1–NSP5 genomic segments of strain A75 are LC834027–LC834037, respectively.

## Results

### Nucleotide sequencing and determination of the genotype constellation

Previously, PCR-based genotyping indicated that strain A75 possesses the G8 genotype, but the VP4 genomic segment of this strain could not be genotyped using VP4-specific primers (for P[4], P[6], P[8], P[9], and P[10]) [65]. Illumina MiSeq next-generation sequencing produced 6.5 × 10^5^ reads (~ 148 bp average length) for strain A75, yielding complete or nearly complete nucleotide sequences of all 11 genomic segments. A summary of nucleotide and deduced amino acid sequence lengths, along with read coverage information, is provided in Additional file 1.

The genotype constellation of the 11 genomic segments of strain A75 was characterized as G8-P[14]-I2-R2-C2-M2-A11-N2-T6-E2-H3 (Table 1). Following the naming convention proposed by the RCWG [24], which emphasizes the G and P genotypes, strain A75 was designated as RVA/Human-wt/KEN/A75/2000/G8P[14]. A comparison of the genotype constellation of strain A75 with those of other G8 and non-G8 strains is shown in Table 1. Across the world, its genotype constellation is shared with several artiodactyl and human strains, including the other African G8P[14] strains, ma31 and AS970, and it closely resembles those of several other artiodactyl and human strains by differing only in NSP1 (A3 or A13 instead of A11), NSP3 (T7 or T9 instead of T6), or NSP4 (E12 instead of E2) genotypes. Human strains, PA169 (G6P[14]), MG6 (G6P[14]), B10925 (G6P[14]), Hun5 (G6P[14]), BP1879 (G6P[14]), EGY3399 (G6P[14]), 111-05-27 (G6P[14]), M0084 (G6P[14]), SKT-27 (G6P[14]), B12 (G8P[1]), 182-02 (G8P[14]), BP1062 (G8P[14]), PR1300 (G8P[14]), 2009726790 (G8P[14]), PR1973 (G8P[14]), ma31 (G8P[14]), BA01 (G8P[14]), BA02 (G8P[14]), 12597 (G8P[14]), PR457 (G10P[14]), and V585 (G10P[14]), are considered—based on their full genome analyses—to possess artiodactyl genomic backbones, and zoonosis has been assumed [9, 25, 37, 39–42, 44, 47, 57, 74–77].

**Table 1:**
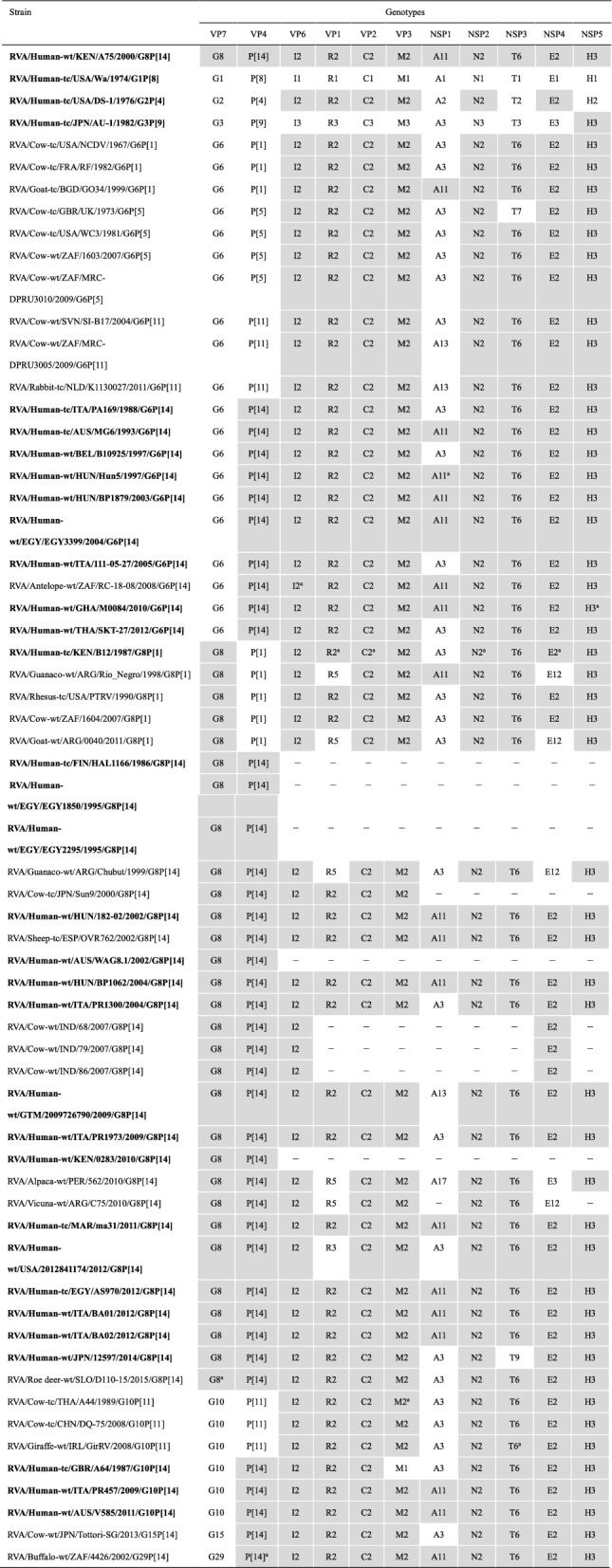
Genotypes of the 11 gene segments of Kenyan G8P[14] strain A75 compared with those of selected human and animal strains with known genomic constellations

Human strains are shown in bold. Gray shading indicates the genomic segments with genotypes identical to those of strain A75

− indicates that no sequence data were available in the DDBJ and EMBL/GenBank data libraries

^a^The genomic segments that are most similar to those of strain A75

### Phylogenetic analyses

Strain A75 was further characterized by constructing phylogenetic trees using the obtained nucleotide sequences for each of its 11 genomic segments, to gain a better understanding of their evolution [9, 57, 78]. The nucleotide sequence identities between strain A75 segments and top matches in closely related strains are shown in Table 2.

**Table 2:** Nucleotide sequence identity between Kenyan G8P[14] strain A75 and close strain(s) in each genomic segment

Genomic segment	Strains that exhibit close nucleotide sequence identities	Identity (%)	DDBJ and EMBL/GenBank accession number	References
VP7	RVA/Roe deer-wt/SLO/D110-15/2015/G8P[14] RVA/Sheep-tc/ESP/OVR762/2002/G8P[14]	97.3 96.8	KY426808 EF554153	[56] [57]
VP4	RVA/Buffalo-wt/ZAF/4426/2002/G29P[14] RVA/Human-tc/GBR/A64/1987/G10P[14]	92.2 91.9	MT234361 EF672563	[79] [80]
VP6	RVA/Antelope-wt/ZAF/RC-18-08/2008/G6P[14] RVA/Cow-wt/ZAF/MRC-DPRU3010/2009/G6P[5]	97.6 97.3	FJ495131 KJ752066	[27] [16]
VP1	RVA/Human-tc/KEN/B12/1987/G8P[1]	97.1	HM627542	[21]
VP2	RVA/Human-tc/KEN/B12/1987/G8P[1]	96.9	HM627543	[21]
VP3	RVA/Cow-tc/THA/A44/1989/G10P[11] RVA/Cow-wt/GBR/R1 WTA11/2013/G6P[11]	96.4 96.3	LC133571 OL988939	[84] [87]
NSP1	RVA/Human-wt/HUN/Hun5/1997/G6P[14] RVA/Human-tc/EGY/AS970/2012/G8P[14]	93.8 92.9	EF554110 KU317461	[57] −
NSP2	RVA/Human-tc/KEN/B12/1987/G8P[1]	98.6	HM627549	[21]
NSP3	RVA/Giraffe-wt/IRL/GirRV/2008/G10P[11] RVA/Roe deer-wt/SLO/D110-15/2015/G8P[14]	96.4 95.8	GQ428138 KY426805	[88] [56]
NSP4	RVA/Human-tc/KEN/B12/1987/G8P[1]	97.5	HM627551	[21]
NSP5	RVA/Human-wt/GHA/M0084/2010/G6P[14] RVA/Cow-tc/CHN/DQ-75/2008/G10P[11]	96.1 96.0	LC460425 GU384198	[76] [89]

− : no reference data were available in the DDBJ and EMBL/GenBank data libraries

**Fig. 1:**
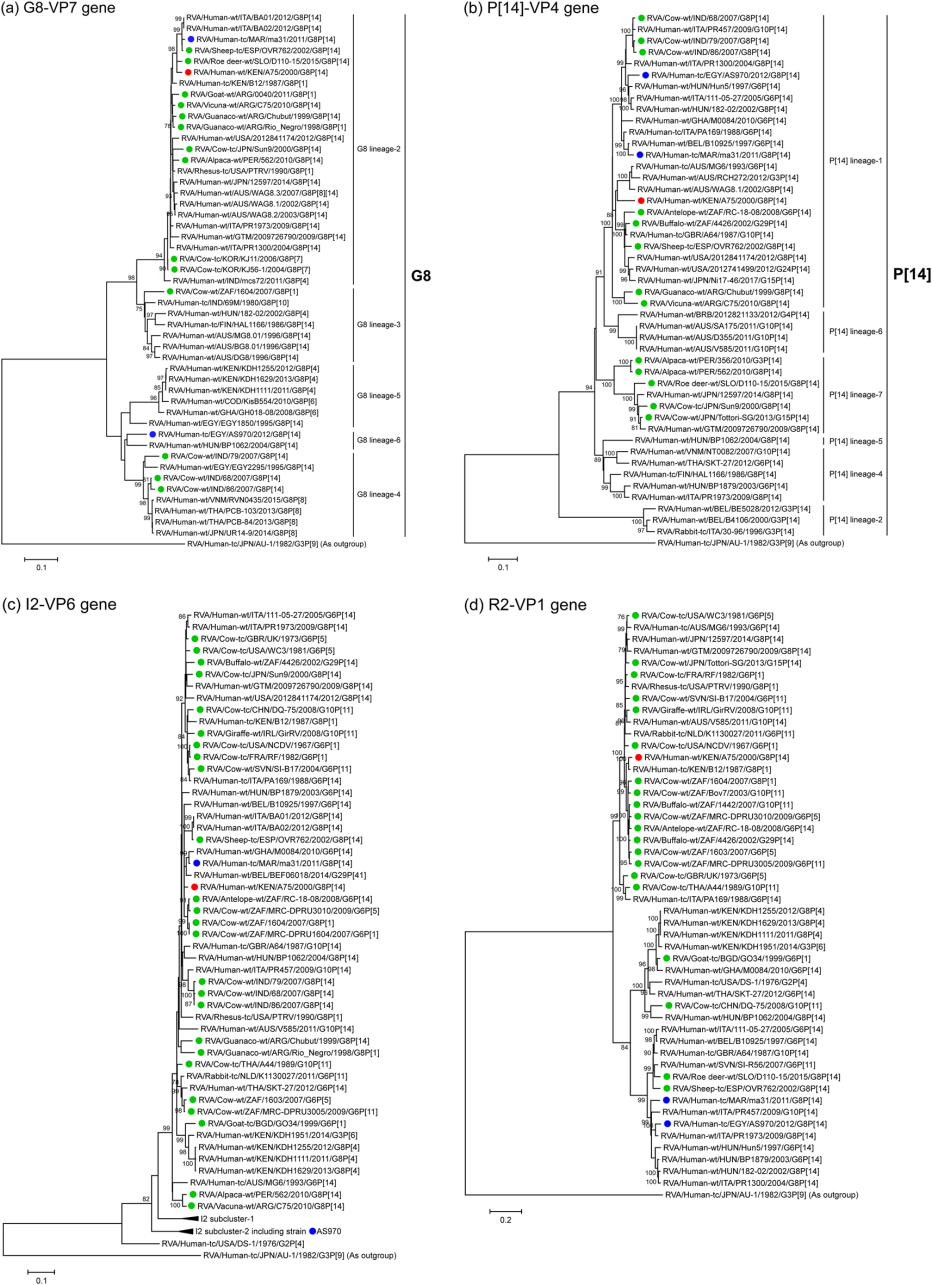

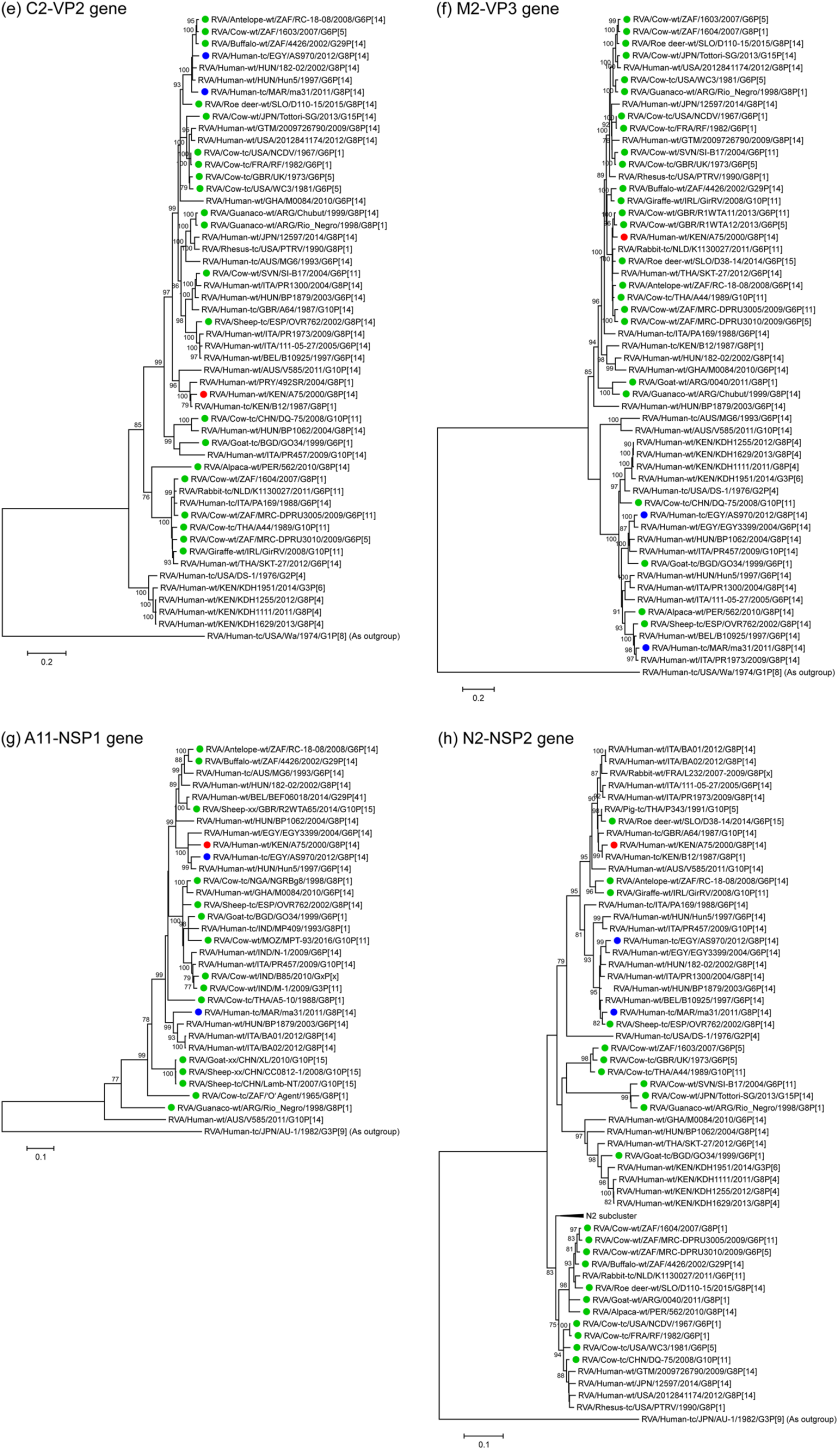

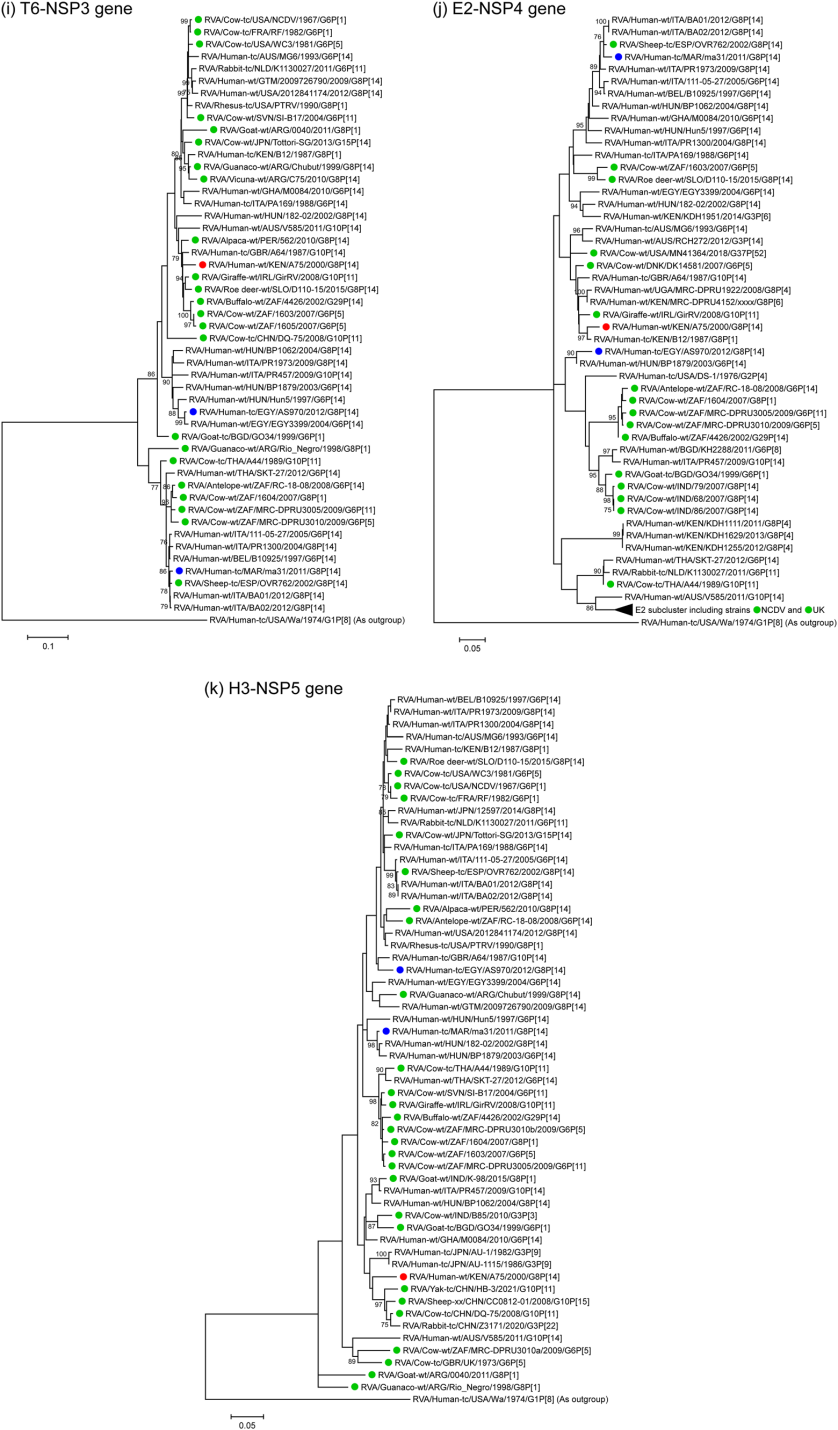
Phylogenetic trees constructed from the nucleotide sequences of the VP7 (**a**), VP4 (**b**), VP6 (**c**), VP1 (**d**), VP2 (**e**), VP3 (**f**), NSP1 (**g**), NSP2 (**h**), NSP3 (**i**), NSP4 (**j**), and NSP5 (**k**) genomic segments of strain A75 and representative RVA strains. The position of strain A75 is indicated by a red circle. Blue circles indicate positions of the two other known human G8P[14] strains, ma31 and AS970, from Africa. Green circles indicate positions of RVA strains isolated from artiodactyls. Bootstrap values of < 75% are not shown. Scale bars: 0.05 (j and k), 0.1 (**a**–**c** and **g**–**i**), and 0.2 (**d**–**f**) substitutions per nucleotide

The VP7 genomic segment of strain A75 showed the highest nucleotide sequence identities, 97.3% and 96.8%, respectively, with that of Slovakian roe deer strain D110-15 (G8P[14]) [56] and Spanish sheep strain OVR762 (G8P[14]) [57] (Table 2). Phylogenetic tree analysis revealed that these three sequences are also closely related to the corresponding sequences of three other artiodactyl-like human strains from Africa and Europe, together forming a cluster supported by high bootstrap confidence (Fig. 1a).

The VP4 genomic segment of strain A75 showed the highest nucleotide sequence identities, 92.2% and 91.9%, respectively, with that of South African buffalo strain 4426 (G29P[14]) [79] and British artiodactyl-like human strain A64 (G10P[14]) [80] (Table 2). Phylogenetically, these sequences clustered together with those of Australian artiodactyl-like human strains, RCH272 (G3P[14]) [81], MG6 (G6P[14]) [57], and WAG8.1 (G8P[14]) [32], and several artiodactyl and artiodactyl-like human strains from South Africa, Europe, the United States, and Japan (Fig. 1b).

The VP6 genomic segment of strain A75 showed the highest nucleotide sequence identities, 97.6% and 97.3%, respectively, with that of South African antelope strain RC-18-08 (G6P[14]) [27] and South African bovine strain MRC-DRPU3010 (G6P[5]) [82] (Table 2). The phylogenetic tree resolution for the highly conserved VP6 genomic segments is not high, and its A75 sequence formed a cluster with those from multiple artiodactyl and artiodactyl-like human strains from Africa and Europe, including the above-mentioned South African strains (Fig. 1c).

The VP1 genomic segment of strain A75 showed the highest nucleotide sequence identity, 97.1%, with that of Kenyan artiodactyl-like human strain B12 (G8P[1]) [21] (Table 2), with which it also formed a phylogenetic tree cluster (Fig. 1d). This cluster was part of a bigger cluster with sequences of several artiodactyl strains from South Africa (Fig. 1d).

The VP2 genomic segment of strain A75 showed the highest nucleotide sequence identity, 96.9%, with that of Kenyan artiodactyl-like human strain B12 (G8P[1]) (Table 2). Phylogenetically, these sequences formed a cluster together with that of Paraguayan artiodactyl-like human strain 492SR (G8P[1]) [83], while the corresponding sequence of Australian artiodactyl-like human strain V585 (G10P[14]) [75] formed a sister group (Fig. 1e).

The VP3 genomic segment of strain A75 showed the highest nucleotide sequence identities, 96.4% and 96.3%, respectively, with those of Thai bovine strain A44 (G10P[11]) [84–86] and British bovine strain R1 WTA11 (G6P[11]) [87] (Table 2). Phylogenetically, these three formed a cluster with sequences from several artiodactyl, artiodactyl-like human, and lapine (rabbit) strains from Africa, Europe, and Thailand (Fig. 1f).

The NSP1 genomic segment of strain A75 showed the highest nucleotide sequence identities, 93.8% and 92.9%, respectively, with those of Hungarian artiodactyl-like human strain Hun5 (G6P[14]) [57] and Egyptian human strain AS970 (G8P[14]) (Table 2). Phylogenetically, these sequences formed a cluster with the corresponding sequence of Egyptian artiodactyl-like human strain EGY3399 (G6P[14]) [77] (Fig. 1g).

The NSP2 genomic segment of strain A75 showed the highest nucleotide sequence identity, 98.6%, with that of Kenyan artiodactyl-like human strain B12 (G8P[1]) (Table 2). Phylogenetically, these two sequences formed a subcluster in a larger cluster that also included sequences of several artiodactyl, artiodactyl-like, and lapine strains from Europe and Thailand (Fig. 1h).

The NSP3 genomic segment of strain A75 showed the highest nucleotide sequence identities, 96.4% and 95.8%, respectively, with those of Irish giraffe strain GirRV (G10P[11]) [88] and Slovakian roe deer strain D110-15 (G8P[14]) (Table 2). Phylogenetically, these three sequences clustered together with those of several artiodactyl and artiodactyl-like human strains from different parts of the world (Fig. 1i).

The NSP4 genomic segment of strain A75 showed the highest nucleotide sequence identity, 97.5%, with that of Kenyan artiodactyl-like human strain B12 (G8P[1]) (Table 2). Phylogenetically, these two sequences formed a subcluster in a large cluster together with sequences of several artiodactyl and artiodactyl-like human strains from Africa, Europe, the United States, and Australia (Fig. 1j).

The NSP5 genomic segment of strain A75 showed the highest nucleotide sequence identities, 96.1% and 96.0%, respectively, with those of Ghanaian artiodactyl-like human strain M0084 (G6P[14]) [76] and Chinese bovine strain DQ-75 (G10P[11]) [89] (Table 2). Phylogenetically, these three sequences formed a cluster, although with low bootstrap confidence, with sequences of several artiodactyl and artiodactyl-like strains from Asia and Europe (Fig. 1k).

**Fig. 2:**
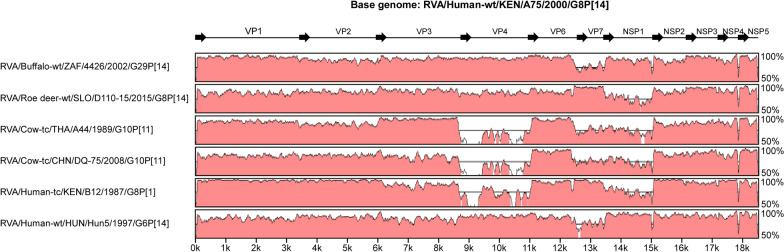
Nucleotide sequence comparison using VISTA similarity plots of the concatenated genome of strain A75 with those of genetically related artiodactyl strains (buffalo 4426, roe deer D110-15, and bovine A44 and DQ-75), and artiodactyl-like human strains (B12 and Hun5). Strain names are listed on the left, and the positions of the 11 genes are indicated at the top. The bottom scale denotes distance in kb. The sequence-based percent identity between study strain A75 and the respective reference strains is indicated on the right. Shading represents the level of conservation. Regions with low similarity at the start and end of individual genomic segments likely indicate missing or divergent sequences in the reference strains compared to the study strain A75

Thus, sequence similarity levels (Table 2 and Fig. 2) and phylogenetic tree analyses (Fig. 1) revealed that all 11 genomic segments of strain A75 are most closely related to artiodactyl and/or artiodactyl-like strains. Notably, five segments—VP7, VP4, VP6, VP3, and NSP3—showed the highest sequence similarity levels with artiodactyl RVA strains, while five other segments—VP1, VP2, NSP2, NSP4, and NSP5—showed the highest similarities with other human RVA strains from Africa. Thus, the genomic makeup of strain A75 highlights the importance of both locality and interspecies transmission in RVA evolution in Africa. In Fig. 2, there appeared to be a region of low sequence similarity in the second half of the NSP5 gene of strain A75 to those of the genetically related artiodactyl and artiodactyl-like human strains. The low similarity of this region was borne by the diversity of sequences in this part of NSP5 genes among these strains with the H3 genotype, but not by any insertions or deletions that are characteristics of strain A75 (data not shown).

## Discussion and conclusion

We determined the full genome sequence of an African HuRVA strain, A75, characterized by the uncommon G8P[14] genotype (RVA/Human-wt/KEN/A75/2000/G8P[14]), isolated from a diarrheic child in Kenya. To the best of our knowledge, our analysis of strain A75 makes it only the second African and the first East African human G8P[14] strain whose full genome has been analyzed and discussed. Strain A75 exhibits a unique genogroup constellation, G8-P[14]-I2-R2-C2-M2-A11-N2-T6-E2-H3, with the non-G/P genotype constellation, I2-R2-C2-M2-A11-N2-T6-E2-H3, commonly found among RVA strains from artiodactyls. Phylogenetic analysis suggests that all 11 genomic segments of strain A75 had an artiodactyl origin at some point in their evolution. Notably, in East Africa (Uganda), the prevalences of RVA infection in cattle and goats are as high as 21% and 9%, respectively [90] and artiodactyl-like human strains have been reported in Kenya and Uganda [9, 91], supporting the possibility and occurrence of artiodactyl-to-human zoonotic transmission events.

Most of the 11 genomic segments, except for VP7, VP6, and NSP1, of the studied Kenyan G8P[14] strain, A75, are only distantly related to those of the other African artiodactyl-like G8P[14] strains, Moroccan ma31 and Egyptian AS970, highlighting the diversity of zoonotic G8P[14] strains in Africa. These observations suggest multiple independent artiodactyl-to-human interspecies transmission events of artiodactyl G8P[14] strains into humans on the African continent. Notably, four of the 11 genomic segments of strain A75—VP1, VP2, NSP2, and NSP4—exhibit a close relationship with those of Kenyan artiodactyl-like human strain B12, pointing to a shared precursor in Kenya and a history of reassortment events.

This study contributes to the expanding awareness of zoonotic transmission from animals to humans. The artiodactyl origin of strain A75 suggests spillover infection of animal strains to human beings due to the close vicinity between humans and artiodactyls, which is still very common in developing countries such as Kenya. The population of Kiambu County, where the child from whom strain A75 was identified lived, practices widespread livestock rearing. Additionally, the county borders a number of counties with game reserves, namely, Nairobi (Nairobi National Park), Kajiado (Amboseli National Park), Nakuru (Nakuru National Park), Nyandarua (Aberdare National Park), and Machakos (Ol Donyo Sabuk National Park). This unique setting fosters frequent human–livestock–wildlife interactions, which might explain the zoonotic precursor of strain A75.

The evolutionary trajectory of strain A75 may be more intricate than a straightforward zoonotic transfer of an artiodactyl strain to humans. It likely involved multiple reassortment events that facilitated the integration of genomic segments from artiodactyl-like strains into human-infecting strains. This is suggested by six of the 11 genomic segments of strain A75—VP1, VP2, NSP1, NSP2, NSP4, and NSP5—displaying higher sequence similarity to artiodactyl-like human strains than to strains isolated from artiodactyls.

The rare detection of the atypical G8P[14] genotype in just one out of 285 diarrheic children during a Kenyan HuRVA strain surveillance program [65], and its absence in 1/614 [45], 0/749 [92–94], and 0/605 [95] cases in later Kenyan HuRVA surveillance efforts, suggests that G8P[14] strains may not yet be fully adapted for efficient human-to-human transmission in Kenya. This hypothesis is supported by the results of amino acids alignments of RVA proteins (VP7, VP4, VP6, VP1–VP3, and NSP1–NSP5), in which any amino acids mutations common among the “humanized” artiodactyl strains such as study strain A75 were not identified in comparison to the “true” artiodactyl strains (Additional file 2). However, as artiodactyl-like G8P[14] strains sometimes cause infections in humans globally, systematic RVA monitoring in both human and animal populations remains critical to further elucidate their potential for sustained human transmission.

Finally, it should be noted that G8P[14] strains share neither the G nor P genotype with ROTAVAC® (G9P[11]) HuRVA vaccine strain, currently used in Kenya. In view of this, the current vaccine may not be able to effectively prevent infection with this zoonotic G8P[14] strain. At present, along with strain A75, only two other human G8P[14] strains from Africa, AS970 and ma31, have full genome sequences available in the DDBJ and EMBL/GenBank data libraries. Continued sequencing efforts will be crucial to further understanding the spread and evolution of African G8P[14] strains.

## Supplementary Information

Additional file 1. Sequence data for the 11 genomic segments of Kenyan G8P[14] strain A75

Additional file 2. Multiple alignments of the amino acids sequences of VP7, VP4, VP6, VP1, VP2, VP3, NSP1, NSP2, NSP3, NSP4, and NSP5of artiodactyl, artiodactyl-like human, and human RVA strains

## Data Availability

The nucleotide sequence data obtained in this study have been deposited in the DDBJ and EMBL/GenBank data libraries. The accession numbers for the nucleotide sequences of the VP1-VP4, VP6, VP7, and NSP1-NSP5 genomic segments of strain A75 are LC834027-LC834037, respectively.

## Abbreviations

KEMRI: Kenya Medical Research Institute
NUITM: Nagasaki University Institute of Tropical Medicine
RCGLID: Research Center for GLOBAL and LOCAL Infectious Diseases
RVA: Group A rotavirus
dsRNA: Double-stranded RNA
RCWG: Rotavirus Classification Working Group
HuRVA: Human RVA
BLAST: Basic Local Alignment Search Tool
NCBI: National Center for Biotechnology Information
RIVM: Rijksinstituut voor Volksgezondheid en Milieu
