# Supplementary material for: Full genome characterization of a Kenyan G8P[14] rotavirus strain suggests artiodactyl-to-human zoonotic transmission
Source: Trop Med Health. 2025 Jun 16;53:82. doi: 10.1186/s41182-025-00759-9 (PMC12168323; doi:10.1186/s41182-025-00759-9)
Supplement: Supplementary file 1 — Additional file 1. Sequence data for the 11 genomic segments of Kenyan G8P[14] strain A75 [file 41182_2025_759_MOESM1_ESM.docx]

**Additional file 1**

Sequence data for the 11 genomic segments of Kenyan G8P[14] strain A75.

| Study strain | Total reads* |  | Genomic segment | | | | | | | | | | |
| --- | --- | --- | --- | --- | --- | --- | --- | --- | --- | --- | --- | --- | --- |
|  |  |  | VP7 | VP4 | VP6 | VP1 | VP2 | VP3 | NSP1 | NSP2 | NSP3 | NSP4 | NSP5 |
| RVA/Human-wt/KEN/A75/2000/G8P[14] | 654,454 | Nucleotides; bp  (% coverage of the full-length) | 1,055  (99.3%) | 2,361  (100%) | 1,349  (99.5%) | 3,286  (99.5%) | 2,690  (100%) | 2,586  (99.8%) | 1,559  (98.3%) | 1,047  (98.9%) | 1,066  (99.3%) | 684  (91.1%) | 648  (97.2%) |
|  |  | Deduced amino acids; aa  (% coverage of the full-length) | 326  (100%) | 776  (100%) | 397  (100%) | 1,088  (100%) | 881  (100%) | 835  (100%) | 491  (100%) | 317  (100%) | 310  (100%) | 175  (100%) | 198  (100%) |
|  |  | Reads mapped to gene segment | 12,406 | 20,729 | 7,537 | 46,349 | 40,738 | 39,278 | 20,956 | 16,086 | 18,268 | 6,402 | 2,582 |
|  |  | Maximum depth of reads | 2,469 | 2,802 | 2,756 | 3,273 | 3,498 | 3,108 | 3,209 | 3,644 | 3,678 | 2,866 | 1,260 |

*Sequence reads remaining after adapter trimming (average length was ~148 bp for strain A75).
